# Male Breast Carcinoma in an Elderly Patient: A Rare Presentation and the Importance of Individualized Management

**DOI:** 10.7759/cureus.106033

**Published:** 2026-03-28

**Authors:** Winthrop Pereira, Ravi Krishnappa, Sumith Deep, Ashima Puri

**Affiliations:** 1 General Surgery, JSS Hospital, Mysuru, IND; 2 Surgical Oncology, JSS Hospital, Mysuru, IND

**Keywords:** cancer, chemotherapy, geriatric oncolology, invasive ductal breast carcinoma, male breast carcinoma, modified radical mastectomy, modified radical mastectomy (mrm)

## Abstract

Male breast carcinoma (MBC) is a rare malignancy and often presents at an advanced stage due to low awareness and social stigma. Management is largely extrapolated from female breast cancer and must be individualized, particularly in elderly patients. An 87-year-old male patient presented with a painless left breast lump since three months and an ulcer over the nipple for since one month. Examination revealed a firm retroareolar mass with a healed ulcer over the lower aspect of the nipple and no palpable axillary lymphadenopathy. Imaging suggested a suspicious lesion, and core needle biopsy confirmed invasive ductal carcinoma. Staging workup with fludeoxyglucose-18 (FDG) positron emission tomography-computed tomography (PET-CT) showed no distant metastasis. The patient underwent a modified radical mastectomy with axillary lymph node dissection. Histopathology revealed Grade II invasive ductal carcinoma with nodal involvement (pT4bN1a, Stage IIIB). Immunohistochemistry demonstrated estrogen and progesterone receptor positivity, human epidermal growth factor receptor 2 (HER2) negativity, and a low proliferative index, consistent with a luminal A subtype. Following multidisciplinary tumour board discussion, adjuvant chemotherapy was omitted, considering advanced age and performance status. The patient was treated with tamoxifen and adjuvant chest wall + axillary radiotherapy. At follow-up, he remains disease-free with a good quality of life. This case highlights the importance of early suspicion in male breast lesions and emphasizes individualized management integrating tumor biology, stage, and patient factors.

## Introduction

Male breast carcinoma (MBC) is a rare malignancy, accounting for approximately 0.5-1% of all breast cancers and less than 1% of cancers in men. Approximately 10% of men with breast cancer have a genetic predisposition, with *BRCA2* mutations being the most common (found in about 5-10% of cases) [1]. The incidence has shown a gradual increase over recent decades and is often diagnosed at a more advanced stage compared to female breast cancer, with most patients presenting between 65 and 70 years of age, largely due to lack of awareness and absence of screening programs [2].

Invasive ductal carcinoma constitutes more than 90% of MBCs, while lobular carcinoma is rare due to the absence of well-developed lobular structures [3]. Risk factors include genetic predisposition (particularly *BRCA2* mutations), hormonal imbalance, obesity, radiation exposure, and family history, although many cases occur sporadically. Population-based analyses have demonstrated that approximately 30-35% of male patients present with stage I disease and 40-45% with stage II disease, while a significant proportion present with stage III (approximately 20%) and stage IV disease (5-10%) [4]. Data from Surveillance, Epidemiology, and End Results (SEER) registries further show that nearly half of cases are diagnosed at a regional stage, indicating a higher likelihood of nodal involvement at presentation. This stage distribution reflects delayed diagnosis and contributes to poorer outcomes in men compared to women [5].

Clinically, MBC typically presents as a painless retroareolar mass, often associated with nipple or skin changes in advanced disease [6]. Diagnosis follows the principles of triple assessment. Ultrasonography is often the preferred initial imaging modality in men, while mammography may be used for further characterization [7]. Magnetic resonance imaging (MRI) is not routinely indicated, but recent studies highlight its role in selected cases for assessing disease extent or when conventional imaging is inconclusive [8]. Core needle biopsy remains the gold standard for diagnosis [9]. Most MBCs are hormone receptor-positive, with luminal subtypes predominating, which has important therapeutic implications [10].

Surgical management remains the cornerstone of treatment for localized disease, most commonly involving modified radical mastectomy with axillary staging. Adjuvant therapy decisions are guided by tumor stage, nodal involvement, and receptor status. Current clinical practice guidelines recommend tamoxifen as the first-line endocrine therapy for hormone receptor-positive MBC [6].

We report a case of locally advanced MBC in an elderly patient, highlighting diagnostic challenges and the importance of individualized multidisciplinary management.

## Case presentation

An 87-year-old male farmer presented with a painless lump in the left breast since three months, along with an ulcer over the left nipple since one month. He was a chronic smoker (30 pack-years) with no significant comorbidities or family history of malignancy.

**Figure 1:**
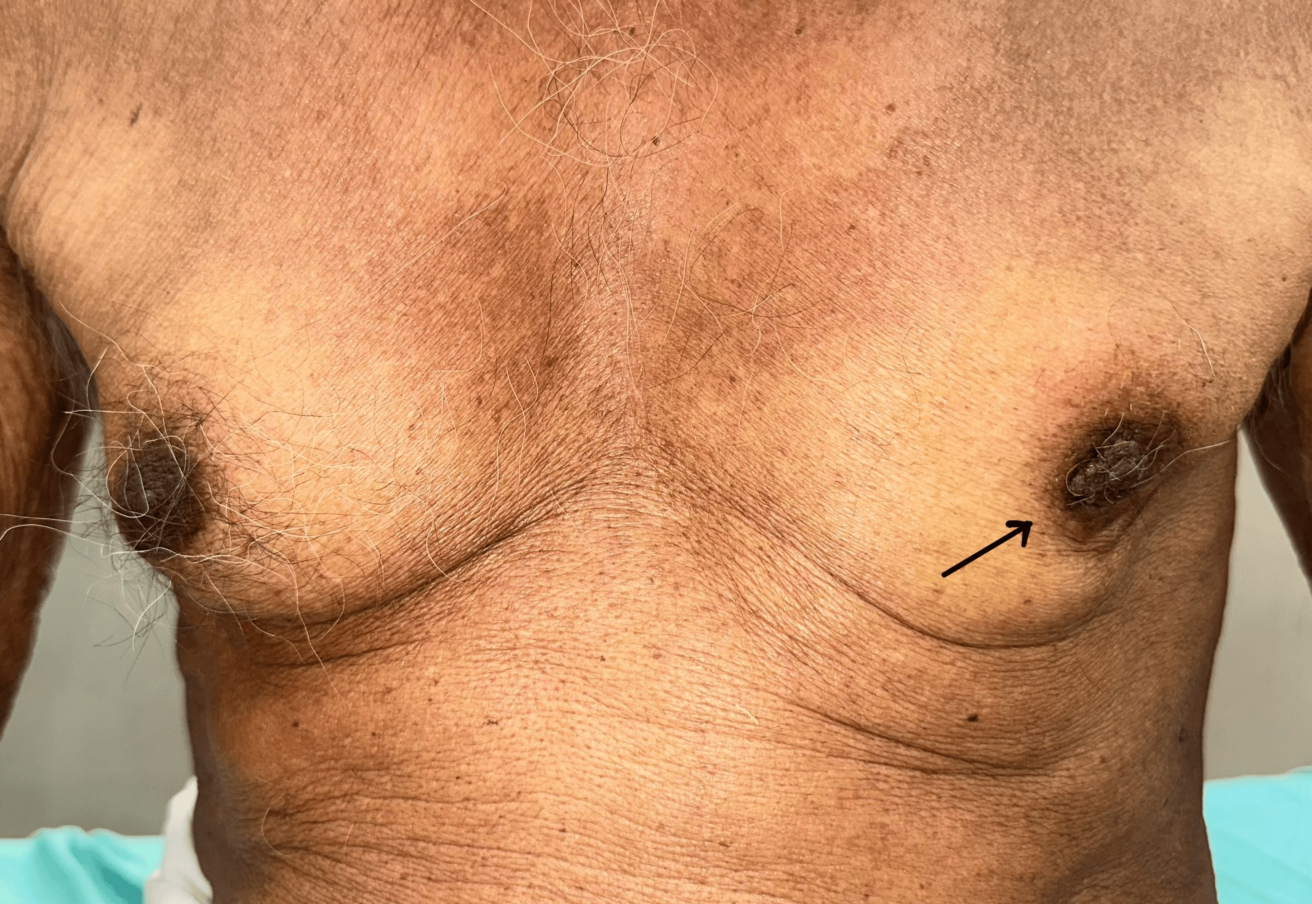
Preoperative comparative clinical photograph showing a normal right breast and a distorted left breast contour (black arrow).

**Figure 2:**
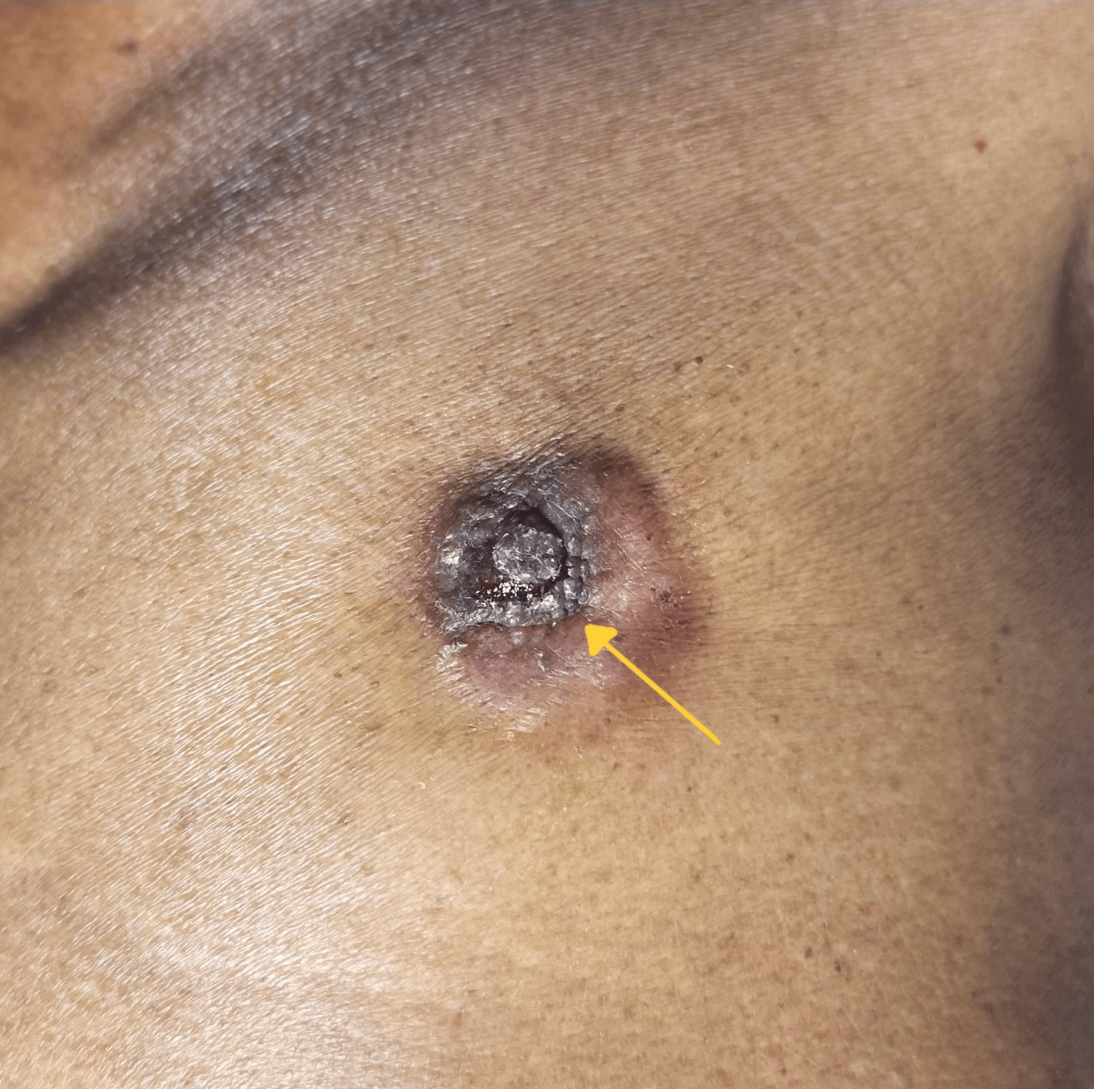
Healed ulcer noted involving the inferior aspect of the left nipple (yellow arrow).

On examination, a firm, non-tender mass measuring approximately 30 × 20 mm was noted in the retroareolar region of the left breast extending toward the lower outer quadrant at the 5 o’clock position (Figure 1). The mass had irregular margins, was fixed to the overlying skin, and was associated with a healed ulcer measuring 20 x 12 mm over the inferior aspect of the nipple, extending from 4 o’clock to 6 o’clock (Figure 2). There was no fixity to the underlying muscle or chest wall. No palpable axillary or supraclavicular lymphadenopathy was detected. The disease was clinically staged as cT4bN0M (American Joint Committee on Cancer (AJCC) Cancer Staging Manual, eighth edition [11]).

Ultrasonography revealed a hypoechoic lesion measuring 34 × 25 mm in the retroareolar region with irregular spiculated margins (Breast Imaging-Reporting and Data System (BI-RADS) 4b), indicating moderate suspicion (10-50%) of malignancy [7]. Axillary evaluation showed no significant lymphadenopathy. Core needle biopsy was done, and findings were consistent with invasive ductal carcinoma, Grade 2 (Nottingham Grading System [9]). Lymphovascular and perineural invasion were not identified due to limited tissue yield.

A whole-body fludeoxyglucose-18 (FDG) positron emission tomography-computed tomography (PET-CT) scan was performed for staging in view of skin involvement, the patient's advanced age, and to avoid multiple sequential imaging studies. Imaging demonstrated a hypermetabolic lesion in the left nipple-areolar complex (standardized uptake value (SUV) ~5) with no evidence of distant metastases.

**Figure 3:**
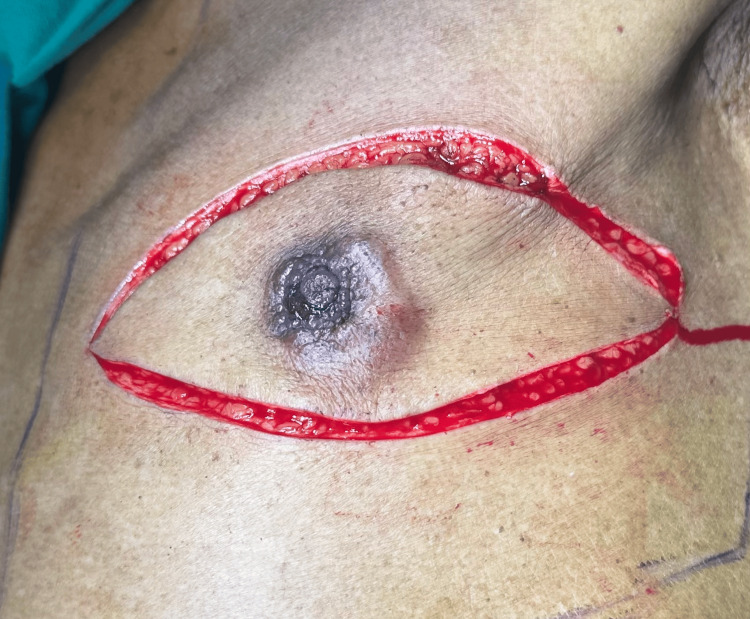
Intraoperative photograph demonstrating the elliptical incision for modified radical mastectomy (MRM) encompassing the nipple–areola complex, with preoperative skin markings outlining the planned mastectomy flaps.

**Figure 4:**
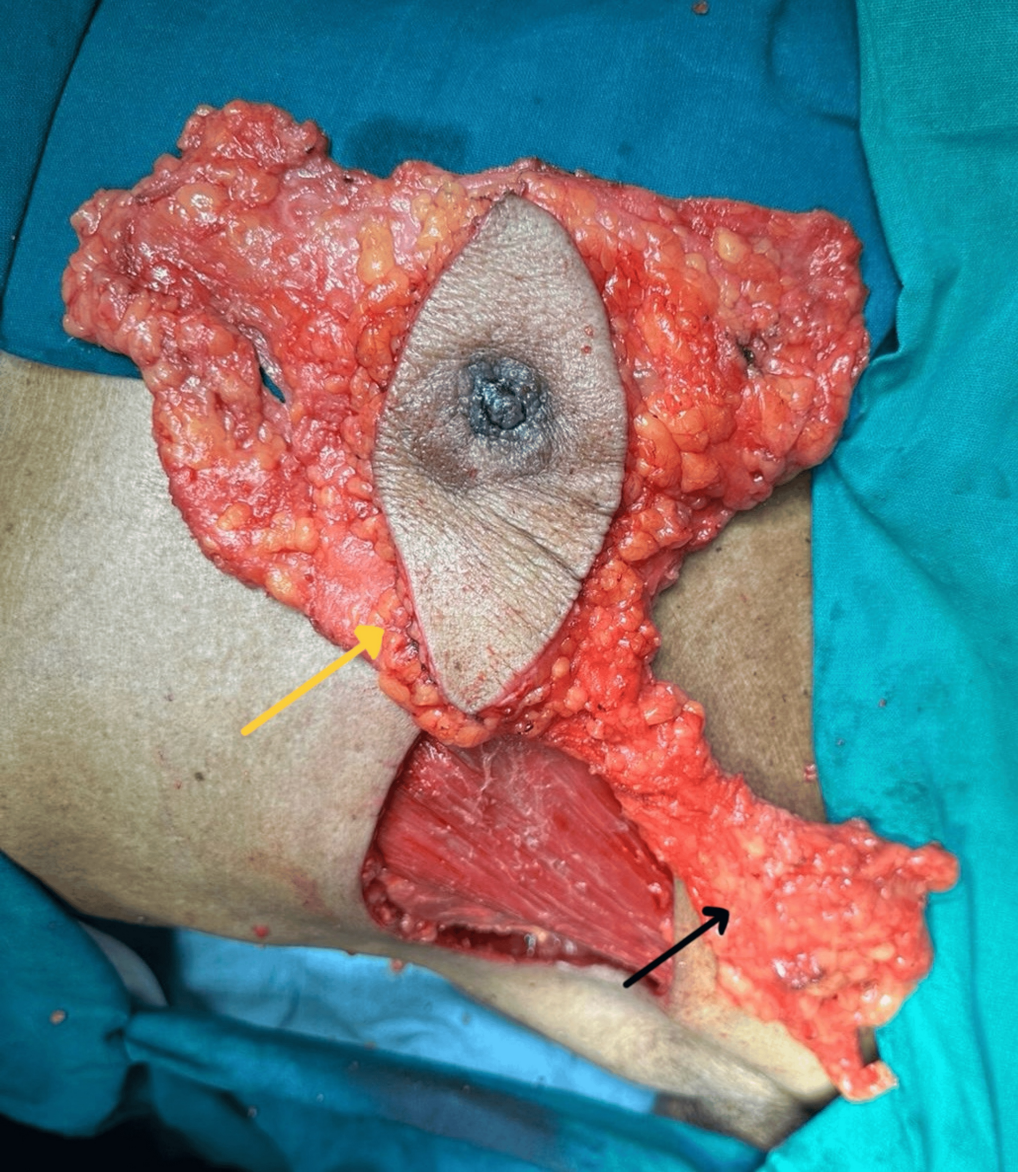
Intraoperative image depicting the mastectomy specimen (yellow arrow) with axillary lymph nodes extracted in toto (black arrow).

The patient underwent a left modified radical mastectomy (MRM) with axillary lymph node dissection. Sentinel lymph node biopsy was not performed due to clinically locally advanced disease (cT4b), and axillary dissection was performed for accurate pathological staging and local disease control (Figures 3, 4).

**Figure 5:**
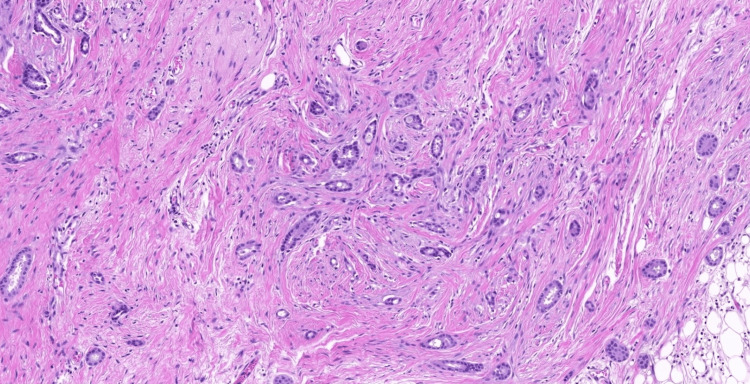
Histopathological section (Hematoxylin and Eosin stain, 10x magnification) showing invasive ductal carcinoma. The image demonstrates nests and cords of pleomorphic epithelial cells infiltrating a dense fibrous stroma, the most common histological subtype found in male breast cancer.

Histopathological examination revealed invasive ductal carcinoma - NST (No Special Type), Nottingham Grade II. The tumour demonstrated focal skin ulceration with dermal extension and desmoplastic reaction (Figure 5). One of 20 dissected axillary lymph nodes showed metastatic involvement. Surgical margins were free of tumor. Lymphovascular and perineural invasion were present. Final pathological stage was pT4bN1a, Stage IIIB (AJCC 8th Edition). Immunohistochemical analysis revealed strong positivity for estrogen receptor (ER) and progesterone receptor (PR), negative expression for human epidermal growth factor receptor 2 (HER2)/neu, and a low proliferative index with Ki-67 of 3-5%, consistent with the luminal A molecular subtype [3]. 

Given the patient’s advanced age and moderate functional status (Karnofsky performance score approximately 70%), the case was discussed at a multidisciplinary tumor board, adjuvant chemotherapy was omitted, and he was started on tamoxifen and received adjuvant chest wall and axillary radiotherapy (50 Gy in 25 fractions).

The patient has been on regular follow-up for six months with periodic clinical evaluation. He expressed satisfaction with the treatment and reported good compliance with tamoxifen therapy, and has completed radiotherapy. He did not develop ipsilateral upper limb lymphedema, and there was no significant impact on daily activities. At the last follow-up, there was no clinical evidence of local recurrence or distant metastasis, and he maintains a stable Karnofsky performance status, which remained at approximately 70% during follow-up. 

## Discussion

MBC presents unique clinical challenges due to its rarity and the lack of prospective data, often necessitating extrapolation from female breast cancer. This is particularly relevant in elderly patients [1-3], as seen in our case.

A hallmark of MBC is delayed presentation. While our patient sought care within three months of noticing a lump, the presence of nipple ulceration (pT4b) at a relatively small tumor size (34 mm) reflects the anatomical lack of breast parenchyma in men, which facilitates early skin and nipple involvement [4].

Imaging plays a crucial role in staging. While mammography is frequently utilized to differentiate gynecomastia from malignancy, it was deferred in this clinical context as the physical findings (nipple ulceration and skin fixation) and sonographic BI-RADS 4b features were already highly suggestive of carcinoma. Similarly, while MRI has an important adjunctive role in assessing chest wall involvement or multifocality, its routine use in MBC is not recommended due to the small volume of male breast tissue [8]. In our patient, conventional ultrasonography provided sufficient clarity regarding the tumor's boundaries and the absence of muscle invasion. Thus, MRI was bypassed to streamline the preoperative phase and avoid unnecessary diagnostic delays in an elderly patient, aligning with National Comprehensive Cancer Network (NCCN) recommendations to use MRI as a problem-solving tool rather than a routine investigation [13].

The presence of nipple ulceration with histologically confirmed dermal invasion established the diagnosis of T4b disease in this patient. Conventionally, T4 breast cancers are considered for neoadjuvant systemic therapy to facilitate downstaging. However, in this case, the tumor was clinically resectable at presentation, with no evidence of fixation to the chest wall or pectoralis muscle [13]. This represents a pragmatic deviation from conventional neoadjuvant strategies in favor of immediate surgical management in a carefully selected patient.

Given the patient’s advanced age, borderline functional status (Karnofsky ~70%), and luminal A tumor biology (low Ki-67, strong hormone receptor positivity), the expected benefit from neoadjuvant chemotherapy was limited, with a higher risk of treatment-related morbidity [13]. Following multidisciplinary discussion, a decision was made to proceed with upfront surgery to achieve timely local disease control, representing an individualized approach in the geriatric setting The use of FDG PET-CT instead of traditional bone scan and CT chest/abdomen represented a tailored approach for this 87-year-old patient. For an elderly patient, PET/CT offers a high-sensitivity "one-stop" staging modality to rule out distant metastasis before committing to radical surgery. This is particularly relevant in locally advanced cases (Stage III), where the risk of occult systemic disease is higher [14].

Surgical management remains the cornerstone of treatment. MRM with axillary lymph node dissection remains the gold standard for T4 male breast cancer. While sentinel lymph node biopsy has gained ground in clinically node-negative (cN0) disease, the presence of T4 disease and the high likelihood of regional spread in MBC (nearly 50% of cases) justified a formal axillary lymph node dissection in this patient to ensure locoregional control and accurate pathological staging [6]. This approach was not intended to obviate the need for postoperative radiotherapy, which was indicated given the presence of skin involvement.

Adjuvant treatment decisions in elderly patients require careful consideration of tumor biology and patient factors. Current guidelines suggest that the benefit of chemotherapy diminishes with advancing age, particularly in luminal A subtypes characterized by low Ki-67 and high hormone receptor expression. The patient's tumor demonstrated a Luminal A profile (ER+/PR+, HER2-, Ki-67 3-5%). These tumors are traditionally less chemosensitive but highly responsive to endocrine therapy [15]. In this patient, advanced age increased the likelihood of chemotherapy-related toxicity, including severe myelosuppression, cardiotoxicity, and neurotoxicity-related fall risks, potentially outweighing the limited absolute survival benefit [16]. Tamoxifen remains the preferred first-line endocrine therapy for MBC. Unlike in postmenopausal women, where aromatase inhibitors are superior, in men, they can lead to a compensatory increase in testosterone and subsequent peripheral aromatization, making tamoxifen the more reliable gold standard for suppressing the hypothalamic-pituitary-testicular axis [3,9]. Post-mastectomy radiotherapy plays a critical role in improving locoregional control in patients with high-risk features such as T4 disease and nodal involvement. However, its application in elderly patients requires careful individualization. Advanced age alone should not be a contraindication; rather, treatment decisions should be guided by functional status, comorbidities, and life expectancy. In older patients, radiotherapy is generally well tolerated, although there is an increased risk of fatigue, skin toxicity, and potential functional decline, particularly when combined with extensive surgery such as axillary lymph node dissection [17]. In our patient, despite advanced age, preserved functional status, and high-risk disease, the use of adjuvant radiotherapy was completed without significant morbidity.

Prognosis in male breast cancer is determined primarily by tumor stage and nodal status. For Stage III disease, the five-year overall survival is 73.8%. While overall survival decreases over time, the 10-year disease-free survival remains high at 81.9%, and the 10-year disease-specific survival is 91.7%. Luminal A tumors, the most common subtype in men (80%), are characterized by high hormone receptor positivity and are associated with favorable long-term outcomes. Consequently, hormonal therapy (specifically tamoxifen) is considered the mainstay of treatment for ER+ cases [18]. 

Our patient’s stable Karnofsky Performance Status (70%) and lack of lymphedema six months post treatment suggest that a de-escalated systemic approach (omitting chemotherapy while maintaining locoregional radiation and endocrine therapy) reflects a balanced approach to oncological control while minimizing treatment-related morbidity.

## Conclusions

MBC in elderly patients presents unique diagnostic and therapeutic challenges, particularly when locally advanced disease necessitates deviation from standard treatment pathways. This case highlights the importance of accurate clinical staging, as well as the need for individualized decision-making in the context of advanced age and functional status. In the context of locally advanced disease with nodal involvement, upfront surgery was undertaken in view of clinical resectability and limited anticipated benefit from systemic chemotherapy. While this approach required comprehensive locoregional treatment, including axillary lymph node dissection and radiotherapy, the patient tolerated therapy without major morbidity and maintained stable functional status. This case underscores the importance of balancing oncological benefit with treatment burden, and emphasizes that management in MBC, particularly in the geriatric population, should be guided by tumor biology, disease extent, and patient fitness rather than protocol alone.
